# Confined Growth by Self‐Combustion of a Cu‐Based Nanophase into Mesostructured Acid Supports for DME Production from CO_2_

**DOI:** 10.1002/cplu.202400760

**Published:** 2025-02-04

**Authors:** Fausto Secci, Valentina Mameli, Marco Sanna Angotzi, Luciano Atzori, Lorenza Piroddi, Nicola Pinna, Mauro Mureddu, Carla Cannas

**Affiliations:** ^1^ Department of Chemical and Geological Sciences University of Cagliari S.S. 554 bivio per Sestu 09042 Monserrato CA Italy; ^2^ Consorzio Interuniversitario Nazionale per la Scienza e Tecnologia dei Materiali (INSTM) Via Giuseppe Giusti 9 50121 Firenze FI Italy; ^3^ Sotacarbo S.p.A. Grande Miniera di Serbariu 09013 Carbonia SU Italy; ^4^ Institut für Chemie and IRIS Adlershof Humboldt-Universität zu Berlin 12489 Berlin Germany

**Keywords:** nanocomposites, mesostructures, self-combustion, CO_2_ conversion, dimethyl ether

## Abstract

This work deals with the design of nanocomposite hydrogenation‐dehydration bifunctional catalysts for the one‐pot conversion of CO_2_ to dimethyl ether (DME), focusing on obtaining a high and homogeneous dispersion of a Cu‐based CO_2_ hydrogenation phase into the pores of mesostructured supports. Particularly, three aluminosilicate mesostructured acid catalysts with catalytic activity towards methanol dehydration and featuring different porous structures (Al‐MCM‐41, Al‐SBA‐15, Al‐SBA‐16) were synthesized and used as supports to host a CuO/ZnO/ZrO_2_ (CZZ) CO_2_ hydrogenation catalyst for methanol synthesis. The use of a mesostructured support allows to maximize the exposed surface of the CO_2_ reduction function by nanostructuring it through its confinement within the mesochannels, thus obtaining nanocomposite bifunctional catalysts with an ultra‐small hydrogenation nanophase. The nanocomposites were obtained using an impregnation strategy combined with a self‐combustion reaction, allowing to incorporate the CO_2_ reduction phase inside the mesopores. In all cases, the characterization shows that the hydrogenation phase species are highly and homogeneously dispersed into the supports as either small nanoparticles or as a nanolayer. The as‐obtained nanocomposites were tested for their catalytic activity and the results discussed taking into account the structural, textural, and acidic properties of the supports and nanocomposites.

## Graphical abstract

Three mesostructured aluminosilicates (Al‐MCM‐41, Al‐SBA‐15, Al‐SBA‐16) with different textural properties are used as supports to obtain bifunctional nanocomposite catalysts for the direct CO_2_‐to‐DME conversion, by hosting a Cu‐based CO_2_ hydrogenation phase incorporated through a self‐combustion impregnation route. A high pore volume and pore size of the support positively affect CO_2_ conversion, while a high surface area positively influences DME selectivity.

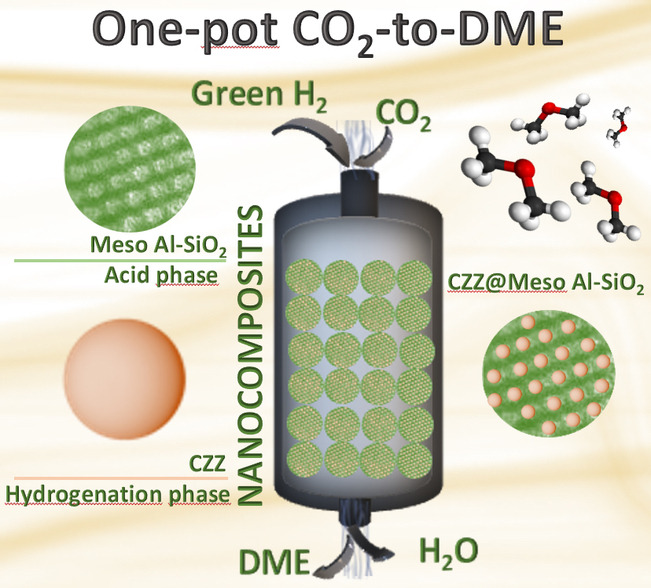

## 1 Introduction

The increasing concern towards the dramatic consequences of global warming has led several governments around the world to adopt various measures in order to reduce CO_2_ emissions. One of these strategies is the Carbon Capture and Utilization (CCU), which consists in the capture of CO_2_ and its transformation into various products such as materials, chemicals and fuels.[^1^, ^2^, ^3^, ^4^, ^5^, ^6^] In a circular economy scenario, CO_2_ captured from flue gases (or even directly from the air), combined with green hydrogen (obtained from water electrolysis using electricity generated from renewable sources), can provide a feedstock to produce the so‐called “green fuels”. This approach is generically named Power‐to‐X, since it consists in using excess electricity produced by renewable power plants to store energy by obtaining various fuels in either liquid (Power‐to‐Liquids) or gaseous (Power‐to‐Gas) form.[^7^, ^8^, ^9^] One of the most promising fuels in this framework is dimethyl ether (DME), a non‐toxic gaseous fuel with chemical‐physical properties similar to those of Liquified Petroleum Gas (LPG); this fact allows to store and transport DME using the same technologies employed for LPG. DME, due to its high cetane number, can be used as an additive for diesel fuel or, after proper modifications to some parts of diesel engines, can completely replace it.[^10^, ^11^, ^12^, ^13^, ^14^, ^15^, ^16^, ^17^, ^18^] CO_2_ is transformed into DME through an initial reduction of CO_2_ to methanol:

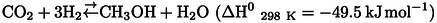




The obtained methanol is subsequently dehydrated to dimethyl ether:

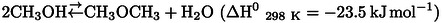




Regarding the first reaction, the catalytic systems reported in the literature mainly consist in Cu‐based CO_2_ reduction/hydrogenation catalysts.[^19^, ^20^, ^21^, ^22^] In these systems, the active phase (Cu) is usually paired with a promoter (ZnO) and a third phase, often ZrO_2_ or Al_2_O_3_, which improves the chemical and thermal stability of the catalyst. The dehydration of methanol to DME, on the other hand, is promoted by solid acidic catalysts, mainly aluminosilicates, like zeolites (mainly ZSM‐5 and ferrierite), and γ‐Al_2_O_3_.[^19^, ^21^, ^22^, ^23^, ^24^, ^25^, ^26^] According to the literature, aluminosilicates are much more active toward methanol dehydration due to the presence of Brønsted acid sites. Brønsted sites, indeed, thanks to their nature, have a significantly higher water resistance,[^27^, ^28^] while Lewis sites, typical of γ‐Al_2_O_3_, are susceptible to deactivation due to the adsorption of the water produced during the reaction.^[21]^

For the DME production from CO_2_, both the two‐step process (in which the two reactions are carried out in separate reactors) and the one‐step process (in which both reactions are performed simultaneously inside the same reactor) have been reported. Due to economic and thermodynamic reasons, however, the one‐step process is preferred; this route, indeed, requires a cheaper equipment (since a single reactor is used) and presents a higher CO_2_ conversion, due to the immediate subtraction of methanol by the dehydration reaction, which shifts the equilibrium.[^19^, ^21^] When the DME production from CO_2_ is carried out with a one‐step process, both the CO_2_ hydrogenation and the dehydration catalysts are needed inside the reactor. The two catalytic functions can be combined by obtaining either a simple physical mixture of the two catalysts or by merging the two phases through chemical routes like coprecipitation or impregnation, leading to an intimate contact between them.[^19^, ^21^, ^22^] In this context, the intimate nature of the contact between the two catalytic functions is believed to positively affect the performances of the catalyst by some authors,[^29^, ^30^] while other researchers observed a detrimental effect.[^31^, ^32^, ^33^, ^34^]

With the aim of developing nanocomposite bifunctional catalysts, in the present work, the mesostructured acidic catalysts presented in^[27]^ were used as supports to host a CuO/ZnO/ZrO_2_ CO_2_ reduction phase using an impregnation process based on a self‐combustion redox reaction. The composite catalysts were then tested for the one‐pot DME production from CO_2_ and their performances were compared considering their structural, textural, and acidic properties. In this context, we chose to use mesostructured materials, considering them ideal to allow the growth of the hydrogenation nanophase inside the confined environment of the mesopores. Indeed, different pore arrangements, sizes, as well as surface areas and pore volumes can be considered key parameters to play with in this synthesis strategy, affecting the structural and morphological features of the final CO_2_ reduction nanophase. On this regard, we already observed that mesostructured materials allow to obtain a homogeneous dispersion of the CO_2_ hydrogenation phase in form of small nanoparticles inside the pores.^[35]^ On the contrary, the use of other systems, like microporous zeolites, would not allow to reach such a fine dispersion of the reduction phase, leading to a deposition located mainly on the external surface of the support, thus not limiting the growth of the metal oxide nanoparticles. The micropores of zeolites like ZSM‐5 (~5.5 Å)[^36^, ^37^] and ferrierite (4.2×5.4 Å)[^38^, ^39^] could indeed only host small metal‐oxygen clusters, being too small to accommodate CuO (PDF card 00–045‐0937), ZnO (PDF Card 00–036‐1451), or ZrO_2_ (PDF card 00–036‐0420; 00–027‐0997) crystal cells. Furthermore, despite the small size of the molecules involved in the reaction, that can easily diffuse even in the micropores of zeolites, the use of mesoporous catalysts, could presumably lead to an improved diffusion of the gaseous reactants. Regarding the synthesis method, approaches like the coprecipitation (the most conventional method to obtain Cu‐based CO_2_ hydrogenation catalysts for methanol synthesis) were considered not appropriate for the purpose, due to the deposition of the hosted phase on the external surface of the support.[^30^, ^40^, ^41^, ^42^] For this reason, we chose to use an impregnation route, particularly the self‐combustion approach since this method allows to obtain various MeO_x_‐based (with M=Cu, Zn, Zr, Ce) nanocomposites supported on mesostructured systems like SBA‐15[^43^, ^44^] and γ‐Al_2_O_3_.^[35]^ This method proved, under certain conditions, to be more effective than the two‐solvent impregnation route,^[44]^ as well as a fast, easy, scalable, and green reaction, which does not require the use of hazardous organic solvents and does not lead to the production of nitrogen oxides during the combustion process.[^45^, ^46^]

## Experimental Section

### Chemicals

The following chemicals were used as received. PEG_20_‐PPG_70_‐PEG_20_ (Pluronic P‐123) average Mn∼5800, Tetraethyl orthosilicate (TEOS) 98 %, and zirconium(IV) oxynitrate ZrO(NO_3_)_2_ 99 % were provided by Aldrich Chemistry; aluminum chloride hexahydrate (AlCl_3_ ⋅ 6H_2_O) 99 %, aluminum isopropoxide>98 %, and copper nitrate hemi‐pentahydrate Cu(NO_3_)_2_ ⋅ 2.5H_2_O 98 % were provided by Alfa Aesar; aluminum nitrate nonahydrate (Al(NO_3_)_3_ ⋅ 9H_2_O)>98 % and hydrochloric acid (HCl) 37 % were provided by VWR BDH Chemicals; nitric acid (HNO_3_)≥65 % and absolute ethanol (CH_3_CH_2_OH) were provided by Honeywell Fluka; hexadecyltrimethylammonium bromide (CTAB)>98 % and glycine (C_2_H_5_NO_2_) 99 % were provided by Sigma; ammonia solution (NH_3_) 28.0–30.0 %, sodium chloride (NaCl) 99 %, and zinc nitrate hexahydrate Zn(NO_3_)_2_ ⋅ 6H_2_O 98 % were provided by Sigma‐Aldrich.

### Synthesis of the Mesostructures

Three different mesostructured aluminosilicates (Al‐MCM‐41, Al‐SBA‐15, Al‐SBA‐16) were synthesized using various sol‐gel routes; particularly, Al‐MCM‐41 was synthesized with a classical sol‐gel synthesis at room temperature, Al‐SBA‐15 was synthesized using a solvothermal method, and Al‐SBA‐16 was obtained from an EISA (Evaporation‐Induced Self‐Assembly) route. The synthetic procedures are described in detail in.^[27]^

### Synthesis of Nanocomposite Catalysts

To obtain the bifunctional nanocomposites the three mesostructured aluminosilicates (Al‐MCM‐41, Al‐SBA‐16, and Al‐SBA‐15) were used as supports; the impregnation was performed following the procedure reported in.[^35^, ^43^] In a typical synthesis, 25 mL of the precursor solution of the CO_2_ reduction phase was first prepared; this solution contained the metal nitrates Cu(NO_3_)_2_*2.5H_2_O, Zn(NO_3_)_2_*6H_2_O and ZrO(NO_3_)_2_ (Cu/Zn/Zr molar ratio=2/1/1.3) with a total metal concentration of 0.90 mol/L. Then, 25 mL of a second solution, containing glycine (1.30 mol/L), was prepared. The two solutions were subsequently mixed to obtain the final nitrate‐glycine solution. After the preparation of the nitrate‐glycine solution, 1 g of the mesostructured support, previously dried overnight at 120 °C, was submerged in 10 mL of the solution into a beaker under vigorous stirring. Water was then let evaporate at 50 °C, until a viscous blue‐greenish gel was obtained. The resulting gel was then sonicated for 5 minutes and submitted to a sudden temperature rise at 300 °C, by putting it into a pre‐heated oven for 1 h. The three nanocomposites were named CZZ@Al‐MCM‐41, CZZ@Al‐SBA‐16, and CZZ@Al‐SBA‐15. The total weight loading of the composites was 33 %.

For comparison purposes, an unsupported CZZ CO_2_ hydrogenation catalyst, with the same composition, was synthesized using the same self‐combustion approach but without a support; the synthesis details are reported in the Supporting Information, including a graphical representation of the synthesis process for both the nanocomposites and the unsupported CZZ (Figure S1).

### Characterization Techniques

Small‐angle X‐ray diffraction patterns were acquired on a 2θ=0.7°–6° range using a Seifert X3000 diffractometer with a θ–θ geometry and a Cu Kα anode (λ=1.5418 Å); wide‐angle X‐ray diffraction patterns were gathered using a PANalytical X'pert Pro equipped with Cu Kα radiation. The value of lattice parameter of the mesostructures was calculated using the equation $a_{0}=\frac{{2d}_{100}}{\sqrt{3}}$ , for the samples with a hexagonal pore structure (Al‐MCM‐41, Al‐SBA‐15, CZZ@Al‐MCM‐41, and CZZ@Al‐SBA‐15);[^47^, ^48^] the formula $a_{0}=d_{110}\sqrt{2}$ was used for the samples with a cubic porous structure (Al‐SBA‐16 and CZZ@Al‐SBA‐16).[^49^, ^50^] Rietveld refinement was performed on the XRD pattern of CZZ using the software MAUD.^[51]^ LaB_6_ from NIST was used as standard reference to determine the instrumental parameters. The CIF structures used for the refinement were 9008961 (CuO), 9007497 (Cu_2_O), 4105681 (Cu), 1011258 (ZnO), 2108450 (ZrO_2_ Baddeleyite), 9009051 (cubic ZrO_2_) from Crystallography Open Database.

Textural characterization was carried out on a Micromeritics ASAP 2020 instrument by determining the nitrogen adsorption–desorption isotherms at −196 °C. Before the analyses, all samples were pre‐treated for 12 h under vacuum at 250 °C (heating ramp, 1 °C min^−1^). The Brunauer–Emmett–Teller (BET) specific surface area (SA) was calculated from the adsorption isotherm in the P/P_0_ range 0.05‐0.17 for Al‐MCM‐41 and CZZ@Al‐MCM‐41; the range 0.05‐0.25 was used for the other samples. The total pore volume (V_p_) was calculated at P/P_0_=0.9975, while mean pore diameter (D_p_) was determined by applying the Barrett−Joyner−Halenda (BJH) model to the desorption branch isotherm for all samples. The pore wall thickness (T_w_) was calculated using the formula$\;T_{w}=a_{0}-D_{p}$ for the samples with a hexagonal mesostructure and $T_{w}=\frac{\sqrt{3}}{2}a_{0}-D_{p}$ for Al‐SBA‐16 and CZZ@Al‐SBA‐16.[^52^, ^53^]

Transmission electron microscopy (TEM), high‐resolution transmission electron microscopy (HR‐TEM), and EDX chemical mapping images were acquired using a field emission gun FEI TALOS F200S microscope at an accelerating voltage of 200 kV. The instrument is equipped with an integrated EDS system with two silicon drift detectors, for qualitative and semi‐quantitative chemical analysis. Line profile EDX characterization was performed on a JEOL JEM 1400‐PLUS microscope operating at an accelerating voltage of 120 kV. The samples were finely ground, and the powders were dispersed in ethanol and sonicated. The resulting suspensions were dropped onto 200 mesh carbon‐coated copper grids; nickel grids were used for the EDX characterization of Cu‐containing samples.

Adsorption microcalorimetry analyses were carried out with a Tian–Calvet heat flow microcalorimeter (Setaram), equipped with a volumetric vacuum line. Before the analysis, each sample (ca. 0.1 g, 40–80 mesh) was thermally pretreated at 250 °C for 12 h under vacuum (5×10^−3^ Pa). Adsorption measurements were then performed by admitting successive doses of pure NH_3_ at 80 °C in order to limit physisorption. The equilibrium pressure relative to each adsorbed amount was measured by means of a differential pressure gauge and the thermal effect was recorded. The run was stopped at a final equilibrium pressure of about 133 Pa. A differential heat (Q_diff_) cut‐off value between specific and non‐specific (physisorption) adsorbent/adsorbate interactions was fixed to 75 kJ/mol, based on adsorption experiments on a previous work in^[27]^ on mesostructured aluminosilicates.

### Catalytic Tests

The catalytic tests on composite catalysts were performed using 1 g of catalyst. In all cases, 2.5 g of α‐Al_2_O_3_, an inert material, were added to the catalytic systems in order to have a total bed volume of ≈3 cm^3^. The resulting gas hourly space velocity (GHSV) was 12000 Ncm^3^ g_cat_^−1^ h^−1^. All catalysts were reduced inside the reactor in a stream of a H_2_/N_2_ mixture (H_2_, 15 vol % in N_2_) at 300 °C for 2 h under atmospheric pressure. The reaction was carried out at 250 °C, 3.0 MPa with a H_2_ and CO_2_ (molar ratio of 3 : 1) mixture for 36 h. The reaction stream was analyzed by a gas chromatograph (Agilent 7890B, Santa Clara, California, CA, USA) equipped with flame ionized detector (FID, for carbon‐containing compounds) and thermal conductivity detector (TCD, for permanent gases), and two columns HP‐Plot Q column (30 m×0.53 mm×40 μm) used to separate and identify CO_2_, methanol, dimethyl ether, C_2_ and C_3_ hydrocarbons and a HP‐Plot Molesieve 5 A (30 m×0.53 mm×50 μm) for H_2_, N_2_, CH_4_ and CO. To avoid condensation of condensable products, connections between the plant gas outlet and GC inlet were heated at 180 °C. Nitrogen was used as an internal standard. CO_2_ conversion (X_CO2_) and products selectivity (S_CO_, S_CH3OH_, and S_DME_), were calculated according to.^[54]^

## 2 Results and Discussion

### 2.1 Structural, Textural, Morphological, and Acid Sites Characterization

**Figure 1:**
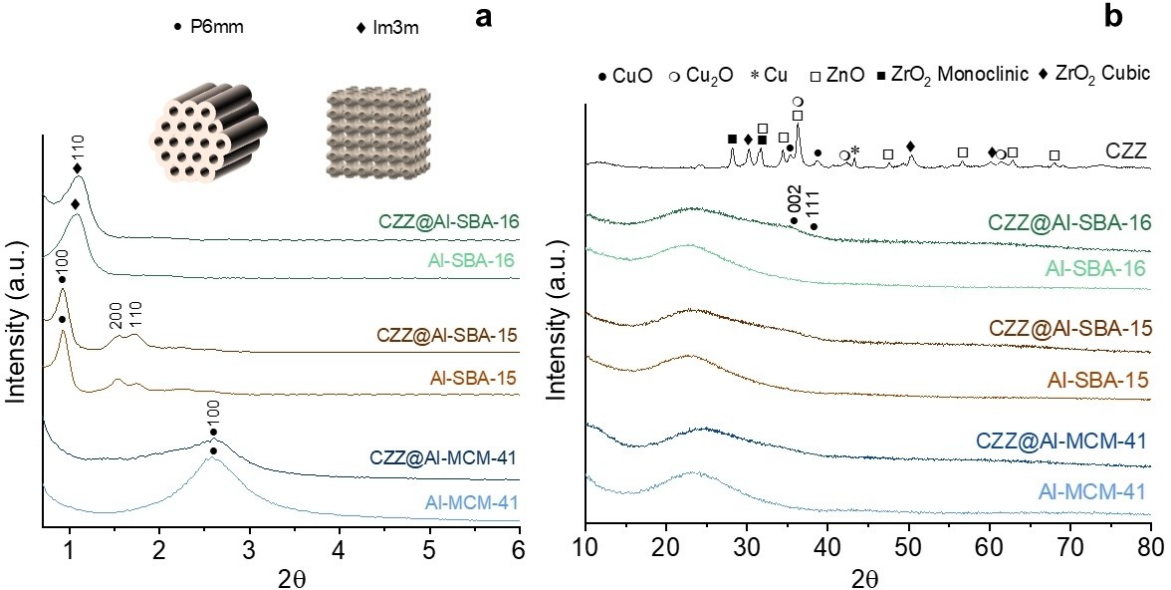
*WA‐XRD (a) and SA‐XRD (b) patterns of composites synthesized by self‐combustion method on Al‐MCM‐41, Al‐SBA‐15 and Al‐SBA‐16 compared with the corresponding mesostructured acidic supports. All the patterns are normalized. Data of the supports added as reference and published in*.^*[27]*^

By the comparison of Small Angle X‐ray diffraction (SA‐XRD) analysis on the bare supports and the nanocomposites it can be observed that the mesoporous arrangement is maintained after the incorporation of the CO_2_ reduction phase inside the mesostructured matrix in all cases, as indicated by the presence of the same bands present in the patterns of the mesostructured supports (Figure 1a). Particularly, a hexagonal (P6mm) pore arrangement was observed for Al‐MCM‐41 and Al‐SBA‐15 and their corresponding composites, while Al‐SBA‐16 and its composite showed a cubic (Im3m) pore arrangement. As shown in Figure S2, where the SA‐XRD patterns are reported with their absolute intensity, a decrease in this value for the main signal of the mesostructure is observed for all composites compared to their corresponding supports, attributable to the presence (33 %) of another non‐mesostructured phase. Moreover, a slight shift towards higher values of 2θ (Figure 1a) is noticeable as a consequence of a decrease in pore diameter associated with the incorporation of the CO_2_ hydrogenation phase inside the mesopores.

Wide‐angle X‐ray diffraction patterns (WA‐XRD) of the synthesized nanocomposites (Figure 1b) do not show any narrow and intense diffraction peak attributable to the CO_2_ reduction phase for any of the samples, differently from what can be observed for the unsupported CZZ catalyst (Figure 1b, Figure S3). However, from their comparison with the XRD pattern of the bare aluminosilicate supports, the presence of a broad and weak band located at 2θ values between 30° and 40° is clearly visible in the case of the nanocomposites. In the WA‐XRD pattern of CZZ@Al‐SBA‐16, where this band is more prominent, its relative maximum is at a 2θ value of 35.5°, which is compatible with the expected position of the main peak (002) of tenorite (CuO). On the other hand, the most intense signal of the unsupported CZZ catalyst falls at a higher value (about 36.3°), corresponding to an overlap of the main signal (111, located at 36.42°) of cuprite (Cu_2_O) with the main signal (101, located at 36.25°) of zincite (ZnO). This finding suggests that the growth within the mesochannels affected both the size and the oxidation state of the Cu in the Cu‐based phase, leading, in the case of nanocomposites, to ultra‐small tenorite nanocrystals, associated with the low and wide signal in the XRD patterns. To further confirm this assumption, it can be observed how the second most intense peak of tenorite (111) is centered at 38.73°, corresponding to the tail on the right of the band (Figure 1b). Furthermore, considering that the shape of the diffraction band of the composites can be justified by the presence of ultrasmall CuO, and no other diffraction signals can be clearly ascribed to ZnO and ZrO_2_, it can be assumed that these metal oxides are either amorphous or low‐crystalline materials. Indeed, although in the 30–40° 2θ range the main reflections of zincite (ZnO) and various ZrO_2_ phases are also located, previous evidence suggested that ZnO has a high affinity for the silica surface, as already observed for ZnO@SiO_2_ ^[55]^ and ZnO@SBA‐15[^56^, ^57^] nanocomposites, thus leading to an amorphous phase. Moreover, since Cu seems to form separate crystalline phases (as observed from the XRD pattern of the unsupported CZZ), and no direct evidences are present for crystalline forms of both ZnO and ZrO_2_ in the nanocomposites, it can be assumed that ZrO_2_ behaves in a similar way to ZnO, and that Cu only forms ultra‐small CuO nanoparticles, confirming the role of the support in the growth of the CO_2_ reduction phase.

As mentioned before, comparing the three composites to each other (Figure 1b), the band attributed to the Cu‐based hydrogenation phase appears to be more visible for CZZ@Al‐SBA‐16, followed by CZZ@Al‐SBA‐15 and CZZ@Al‐MCM‐41, that shows the weakest signal. This finding can presumably be ascribed to the different pore arrangement (cubic *vs*. hexagonal) that, in the case of CZZ@Al‐SBA‐16, allows the growth of the nanoparticles in a three‐dimension porous network, rather than in a 2D one. In this less confined 3D arrangement, indeed, larger nanocrystallites may form. Regarding the Al‐SBA‐15 and Al‐MCM‐41 composites, the slightly more intense diffraction band observed on CZZ@Al‐SBA‐15 can be attributed to the larger pore diameter (Table 1) of Al‐SBA‐15 (7.0 nm) compared with Al‐MCM‐41 (2.1 nm). Furthermore, the other textural properties of the support may play an additional role in determining the final crystallite size of the CO_2_ reduction phase, for instance the higher the surface area and pore volume the finer the dispersion of the hydrogenation phase and the smaller the crystallite size.

**Table 1:** *BET surface area (SA), pore volume (V_p_), mean BJH pore diameter (D_p_), mesostructure lattice parameter (a_0_), and wall thickness of all the samples. Data of the supports added as reference and published in*.^[27]^ *Relative standard deviation: %RSD (SA)=2.1 %; %RSD (Vp)=1.1 %; %RSD (Dp)=1.8 %*.

Sample	SA (m^2^/g)	Loss %	V_p_ (cm^3^/g)	Loss %	D_p_ (nm)	a_0_ (nm)	Tw (nm)
Al‐MCM‐41	1262	54 %	0.77	60 %	2.1	4.0	1.9
CZZ@Al‐MCM‐41	586		0.31		1.7	3.9	2.2
Al‐SBA‐15	673	44 %	1.07	36 %	7.0	11.1	4.1
CZZ@Al‐SBA‐15	376		0.68		6.7	11.0	4.3
Al‐SBA‐16	437	41 %	0.52	37 %	4.6	11.6	5.4
CZZ@Al‐SBA‐16	260		0.33		4.3	11.3	5.4

**Figure 2:**
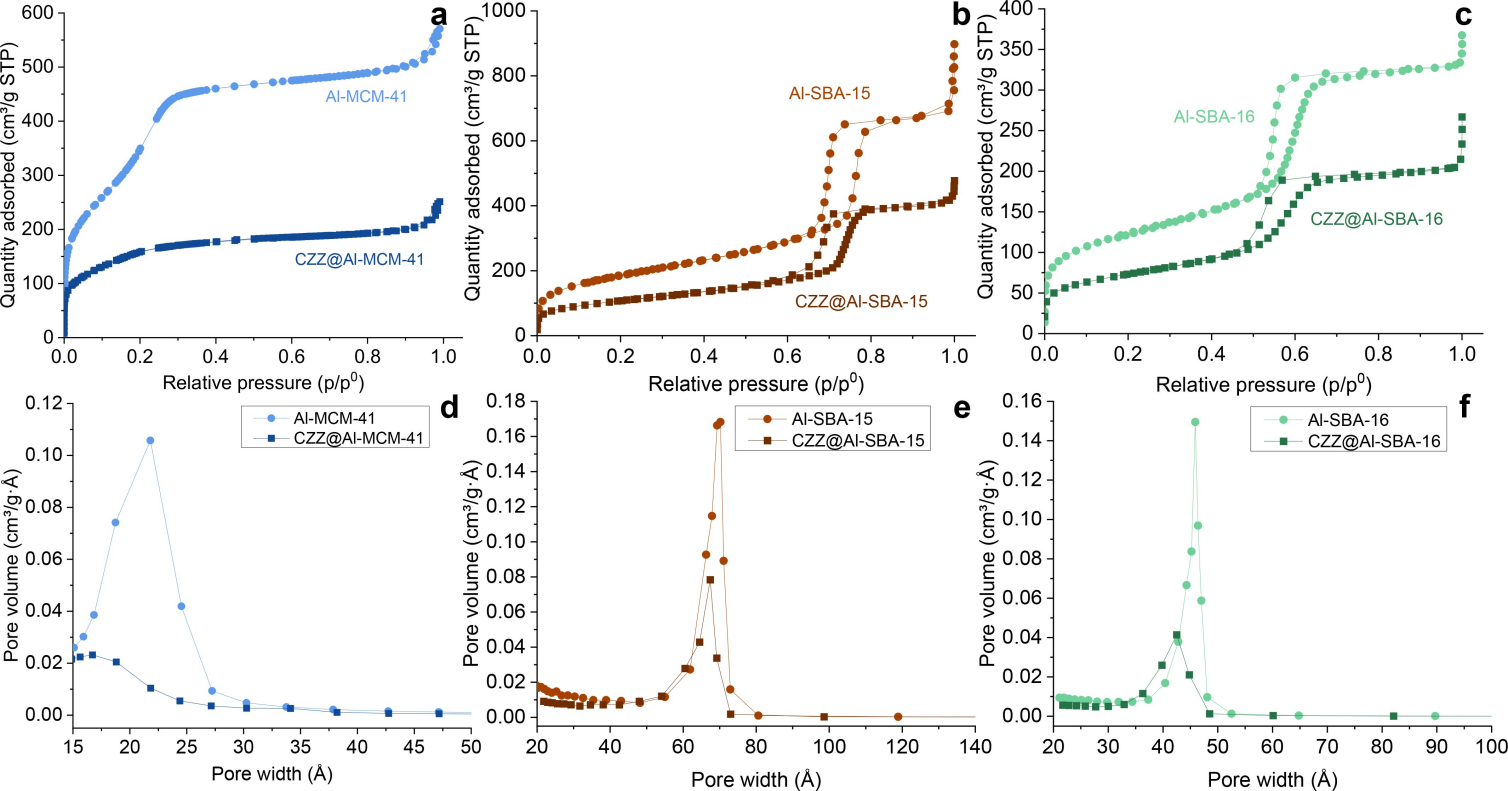
*Nitrogen physisorption isotherms (a–c) and BJH pore size distribution (d–f) of the nanocomposites and their supports. Data of the supports added as reference and published in*.^*[27]*^

Figure 2 shows the nitrogen physisorption isotherms of the three mesostructured aluminosilicates, and the three corresponding nanocomposite samples; textural properties data are reported in Table 1.

Al‐MCM‐41 shows type IVb isotherms with no hysteresis cycle, typical of ordered mesoporous M41S materials^[58]^ (Figure 2a); the isotherms of the corresponding composite (CZZ@Al‐MCM‐41), on the other hand, feature an almost complete disappearance of the capillary condensation branch (Figure 2a), indicating a transition from mesoporosity to microporosity, as expected considering the very small pore diameter of the support (2.1 nm) and the growth of the Cu‐based CO_2_ reduction phase inside the mesochannels. This fact also reflects in the BJH pore size distribution curve (Figure 2d), which exhibits a maximum value below 2 nm, the lower limit of the mesopore range (2–50 nm), in agreement with the shift in terms of 2θ values of the SA‐XRD pattern, indicating a reduction of the pore size towards the micropore range.

On the contrary, both CZZ@Al‐SBA‐15 (Figure 2b) and CZZ@Al‐SBA‐16 (Figure 2c) clearly show that the mesoporosity was maintained after the deposition of the CO_2_ hydrogenation phase. Both the composites feature, indeed, type IVa isotherms with capillary condensation branches and the same hysteresis cycles shown by the corresponding supports. Particularly, they show, respectively, a type H1 and a type H2 hysteresis cycle (IUPAC classification); these curves are typical of mesoporous materials with a narrow distribution of large pores. The type H1 hysteresis cycle is typical of a hexagonal arrangement of cylindrical pores,[^59^, ^60^] while the type H2 cycle is associated with a cubic arrangement of mesopores with necks and cages.[^49^, ^53^, ^61^] The condensation branch is less steep in the composites compared to the supports, indicating a broadening of the pore size distribution due to the functionalization process, also shown by the BJH curve (Figure 2e–f).

In terms of textural properties, a significant decrease in terms of both surface area and pore volume is observed for all composite samples compared to the corresponding supports (Table 1), as expected as a consequence of the inorganic functionalization of the mesopores with the CO_2_ reduction phase. This decrease is also pointed out by the drop of the physisorption isotherms and, consequently, of the BJH pore size distribution curves to much lower values of adsorbed volume (Figure 2). Also, a decrease in terms of mean pore diameter can be observed, attributable to the incorporation of the CO_2_ reduction phase on the inner walls of the mesopores. The pore wall thickness was calculated from the values of lattice parameter and the BJH mean pore diameter, using the formulas reported in the experimental section. The results, reported in Table 1, show a thickening of the mesopore walls due to the impregnation process. Furthermore, the values of surface area and pore volume obtained for the three supports may explain their role in affecting the crystallite size of the CO_2_ hydrogenation phase, as previously hypothesized in the discussion of the WA‐XRD data. In particular, besides the different mesopore framework, Al‐SBA‐16 exhibits the lowest surface area and pore volume thus leading to a lower dispersion of the reduction phase, while the highest surface area of Al‐MCM‐41 and pore volume of Al‐SBA‐15 ensure its finer dispersion and, as a consequence, lower crystallite sizes.

**Figure 3:**
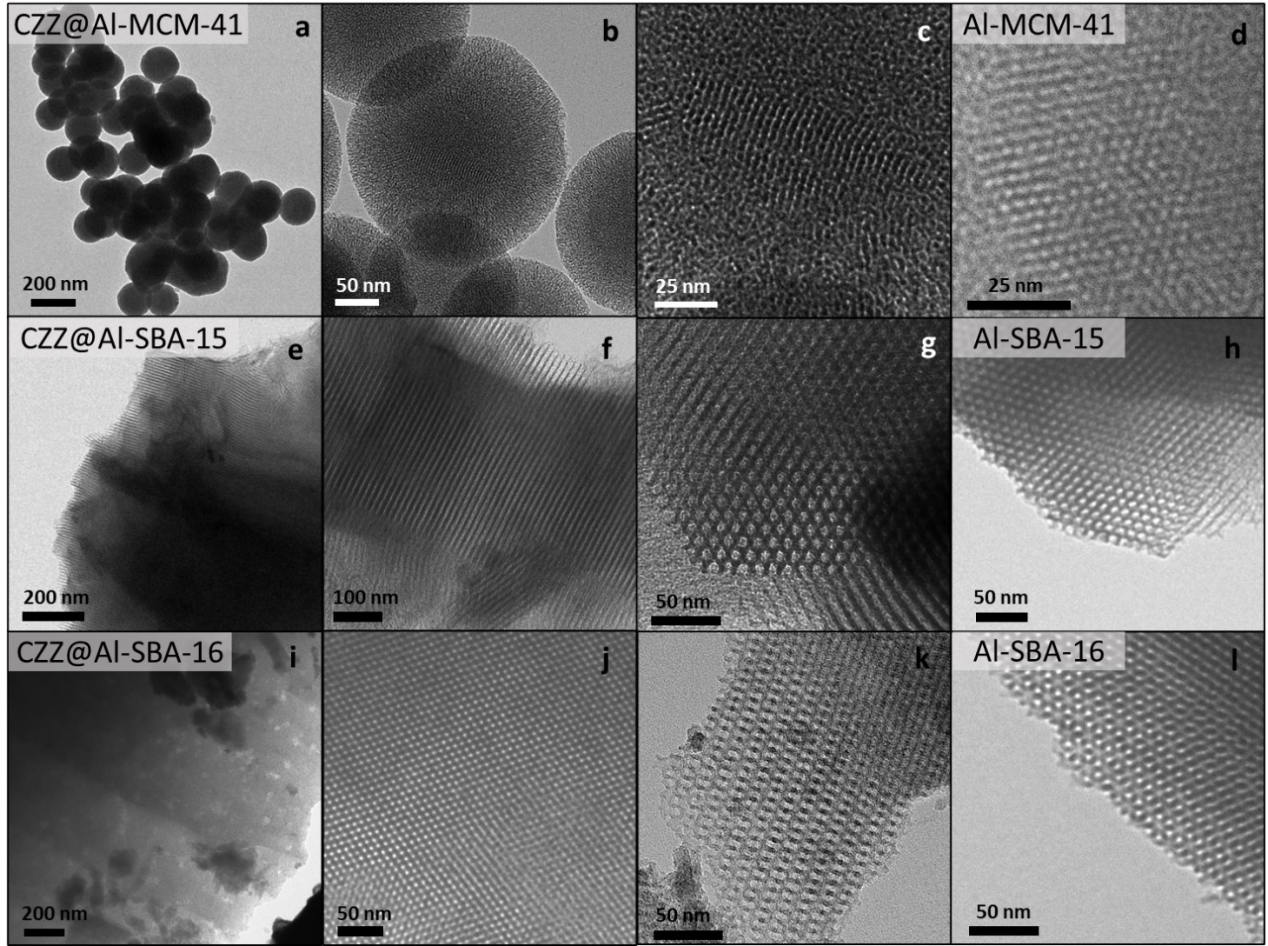
*TEM micrographs of CZZ@Al‐MCM‐41 (a–c), CZZ@Al‐SBA‐15 (e–g), and CZZ@Al‐SBA‐16 (i–k) nanocomposites and their supports Al‐MCM‐41 (d), Al‐SBA‐15 (h), and Al‐SBA‐16 (l). Data of the supports added as reference and published in*.^*[27]*^

**Figure 4:**
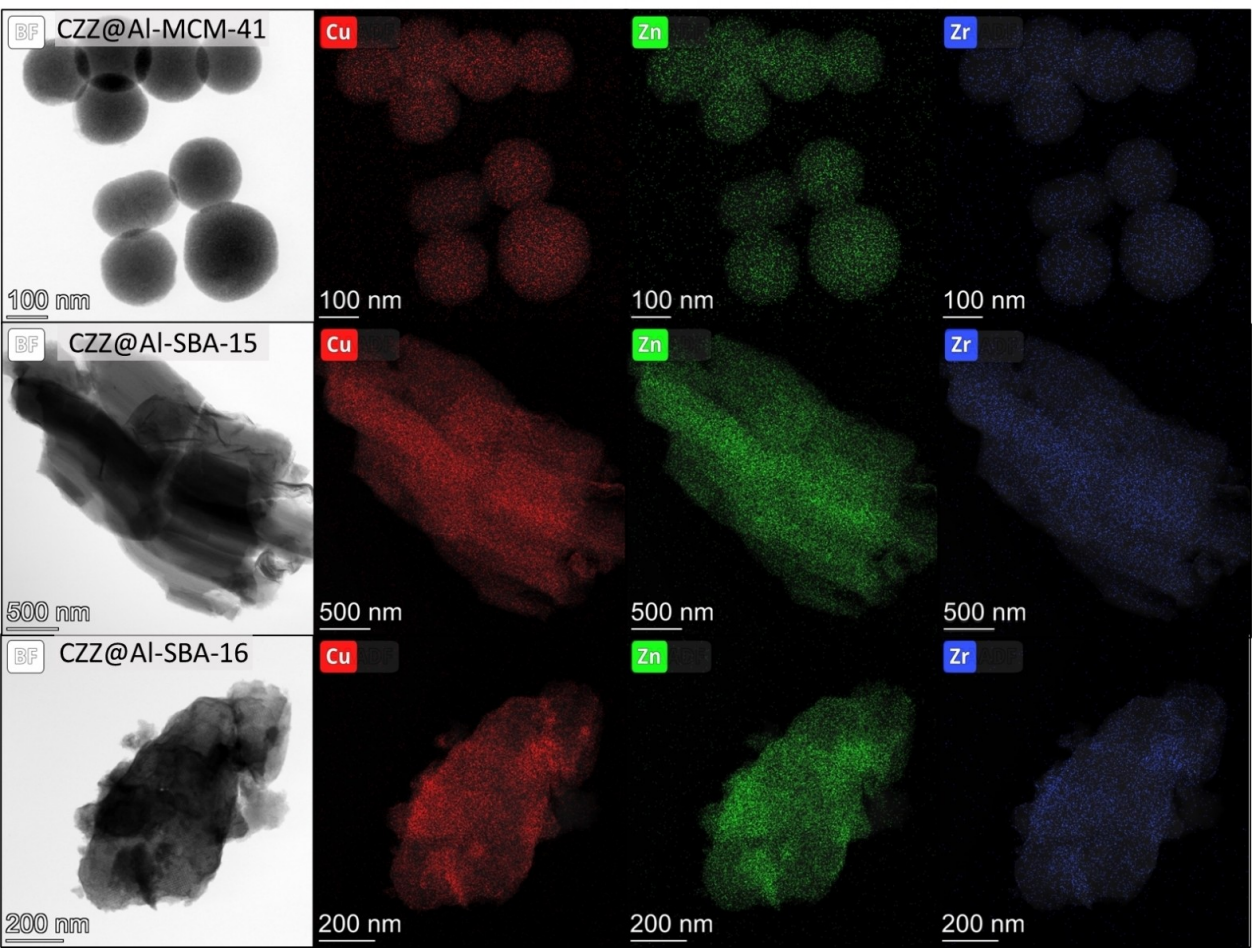
*EDX chemical mapping of CZZ@Al‐MCM‐41 (a–d), CZZ@Al‐SBA‐16 (e–h), and CZZ@Al‐SBA‐15 (i–l) composites*.

TEM imaging (Figure 3) and EDX chemical mapping (Figure 4) of the three nanocomposite catalysts evidence the overall homogeneity of dispersion of the CO_2_ hydrogenation phase throughout the mesostructured matrix with ultra‐nano sizes, as furtherly discussed in detail. These results significantly differ from those of the unsupported CZZ catalyst that, according to the EDX chemical mapping (Figure S5) and line‐profile (Figure S6), features the presence of larger and segregated nanoparticles, indicating that the presence of a support is fundamental to obtain nanocomposites with a homogeneous distribution of the CO_2_ reduction phase. The ordered porous structure is maintained after the impregnation process, as can be seen from the TEM micrographs. The absence of high‐contrast particles attributable to the Cu‐based reduction phase species on the external surface of the support suggests that the reduction phase has been incorporated inside the mesopores in an ultra‐nano form, as already indicated by WA‐XRD analysis. The only noticeable difference between the supports and the nanocomposites is the presence, for the nanocomposites, of some slightly darker spots over the mesostructured matrix, presumably indicating zones with a relatively higher load of CO_2_ hydrogenation phase. This finding is particularly evident in the case of the CZZ@Al‐SBA‐16 nanocomposite, in agreement with WA‐XRD and nitrogen physisorption results. The success of the impregnation route in obtaining a homogeneous distribution of hydrogenation phase is also confirmed by EDX chemical mapping (Figure 4) of Cu, Zn and Zr, showing the lack of large Cu‐, Zn‐, or Zr‐containing particles outside the mesopores of the supports, and furtherly supporting the assumption that those species are homogeneously dispersed inside the pores in the form of either small nanoparticles or as a nanolayer, achieving an intimate mixing of the different CO_2_ reduction species. Owing to intrinsic limits related to TEM imaging, it was not possible to directly observe the presence of nanoparticles of the reduction phase inside the mesopores of the supports. Particularly, this is due to the very small size of the nanoparticles, as also confirmed by the fact that they are not observable by X‐ray diffraction either. Furthermore, the observation of such small particles by TEM requires high magnifications and high voltages (up to 200 kV), and thus a rapid degradation of the sample may occur.

**Figure 5:**
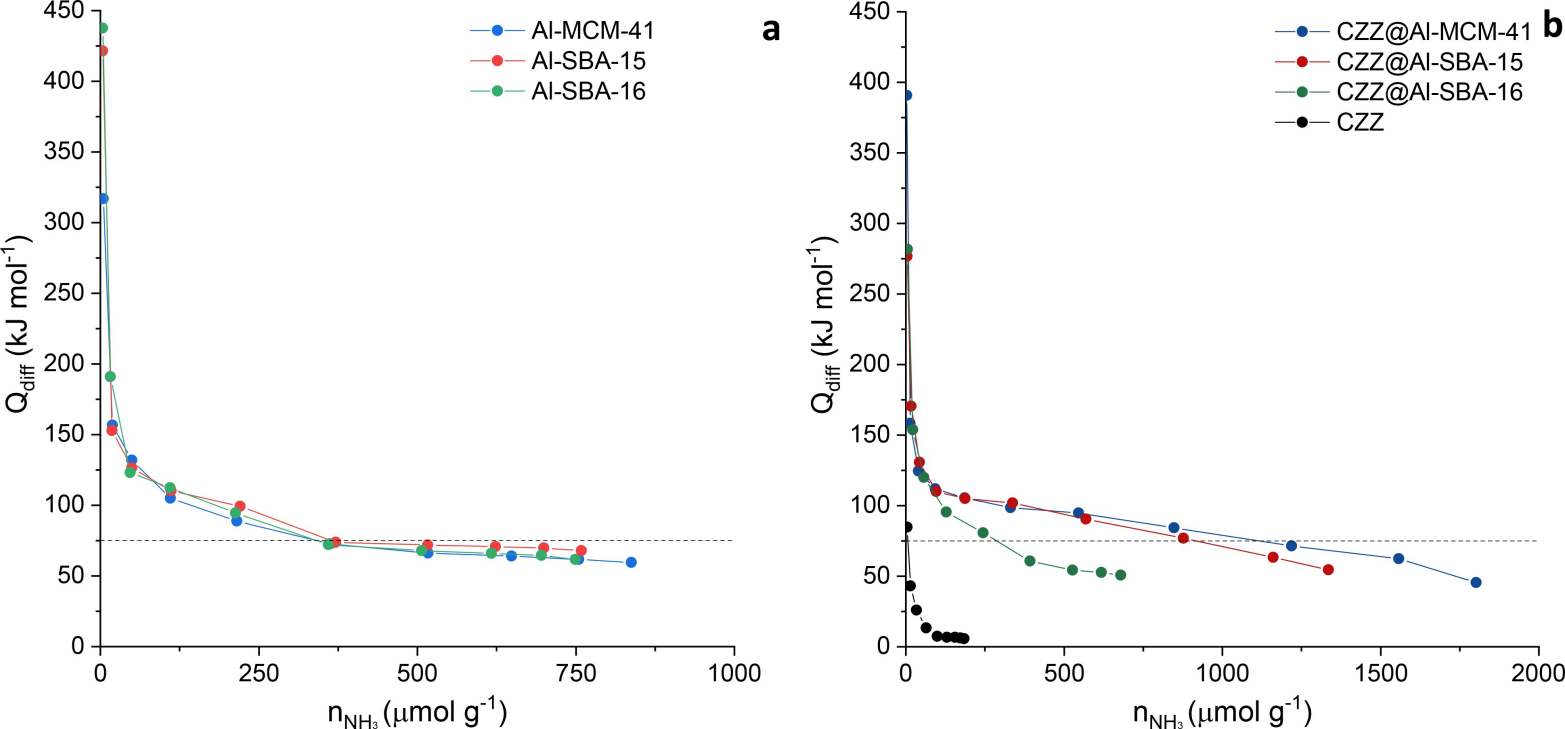
*Differential heat of adsorption (Q_diff_) as a function of the NH_3_ uptake for the mesostructured aluminosilicate supports (a) and for the composite catalysts and unsupported CZZ (b). Data of the supports added as reference and published in*.^[27]^ *The dotted lines represent the cutoff value between physical and chemical adsorption (75 kJ mol^−1^)*.

The NH_3_‐microcalorimetry analysis points out that the functionalization process with the Cu‐based CO_2_ reduction phase led to an increase in the number of acid sites, mainly weak ones, for the composites CZZ@Al‐MCM‐41 and CZZ@Al‐SBA‐15 compared to their corresponding supports (Figure 5, Table S3). This fact might be ascribed to the presence of ZrO_2_ in the reduction phase, which, according to the literature, presents Lewis acid sites.^[28]^ Interestingly, the microcalorimetry analysis on the unsupported CZZ hydrogenation catalyst only shows an extremely low amount of weak acid sites. This finding can be attributed to the formation of ZrO_2_ in the form of much larger nanoparticles (40–50 nm according to Rietveld analysis, see Table S1), leading to a poor dispersion of ZrO_2_ throughout the CZZ mixed‐oxide catalyst with a low surface area. On the other hand, the CZZ@Al‐SBA‐16 composite features a slightly lower amount of acid sites, compared to its support (Figure 5, Table S3). Again, this fact might be related to a possibly lower dispersion of the CZZ CO_2_ hydrogenation phase inside the Al‐SBA‐16 support compared with that of the other nanocomposites, due to its lower surface area and pore volume (as reported in Table 1). Furthermore, the effect of the different mesopore arrangement and pore size on the diffusion of the precursor species during the impregnation process, leading to a different dispersion of the various Cu‐, Zn‐, and Zr‐based phases into each other and throughout the support, cannot be excluded.

### 2.2 Study of the Catalytic Performances and Discussion of the Results

**Figure 6:**
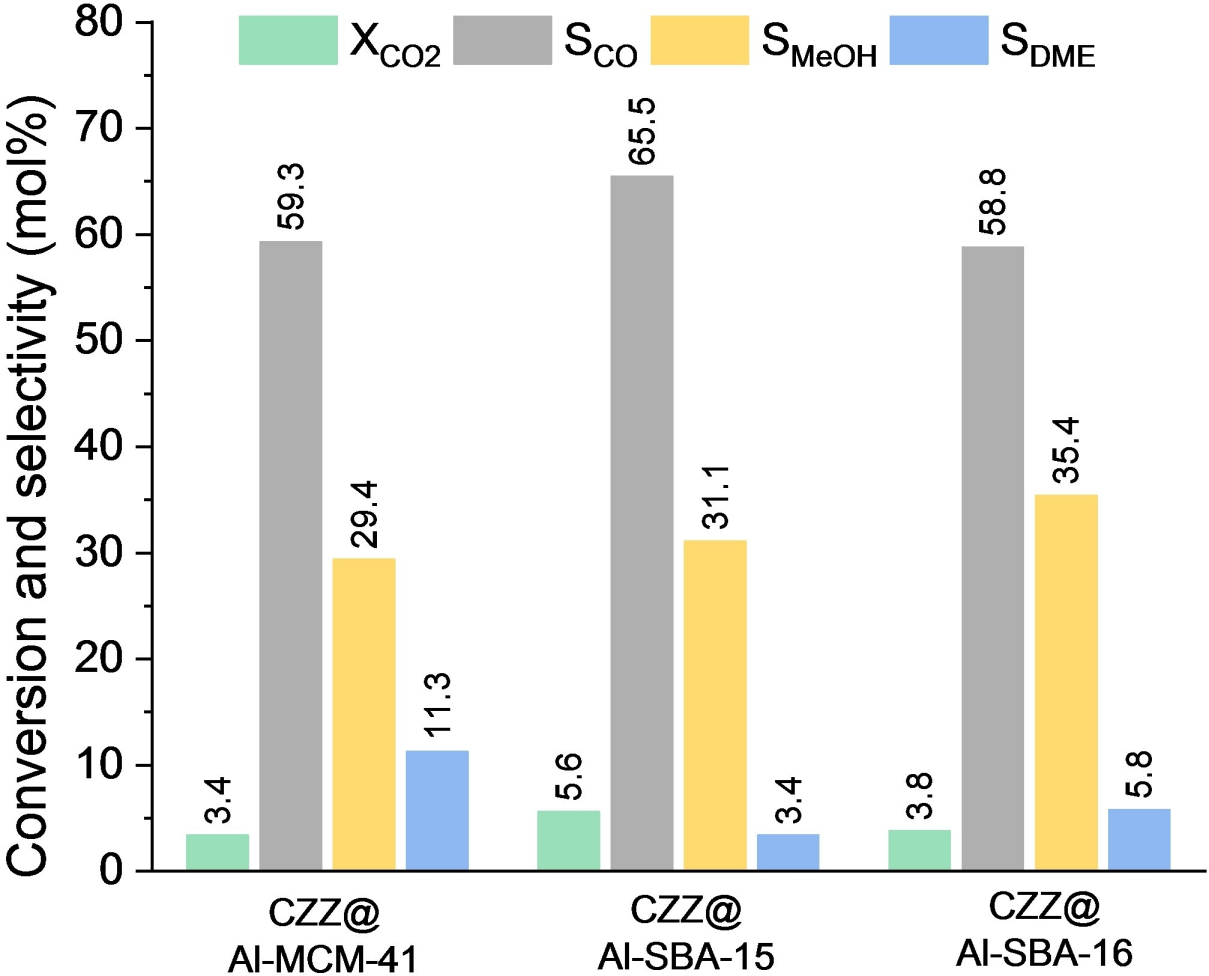
*Mean values of CO_2_ conversion and selectivity to CO, methanol and DME for the nanocomposites*.

All the nanocomposite catalysts were tested for the one‐pot CO_2_‐to‐DME process (Figure 6). The determined performances were compared in order to assess the effect of the different mesostructures and textural properties on the catalytic behavior of both the acid and the CO_2_ hydrogenation catalysts.

As it can be seen in Figure 6, the nanocomposite catalysts feature values of CO_2_ conversion ranging from 3.4 mol % (CZZ@Al‐MCM‐41) to 5.6 mol % (CZZ@Al‐SBA‐15). The higher value of CO_2_ conversion is shown by CZZ@Al‐SBA‐15, presumably because of the high pore volume and pore diameter of the aluminosilicate support (both the highest ones among the three supports; Table 1), that, together with its high value of surface area, allows a better dispersion of the CO_2_ hydrogenation phase, as indicated by both WA‐XRD (Figure 1) and TEM‐EDX (Figure 3). Also in the case of CZZ@Al‐MCM‐41, the textural properties of the acidic support (particularly its high surface area; Table 1) may allow a fine dispersion of the hydrogenation phase. Nevertheless, the lower value of CO_2_ conversion may be ascribed to a possible occlusion of a fraction of the small mesopores (2.1 nm for Al‐MCM‐41 *vs*. 7.0 nm for Al‐SBA‐15), due to the functionalization with the Cu‐based reduction phase. This hypothesis is supported by the more significant drop in terms of surface area (‐54 %) and pore volume (‐60 %) for the Al‐MCM‐41 support after its functionalization, compared to the other support‐nanocomposite pairs that, on the other hand, show very similar drops, as expected taking into account the same weight loading of CO_2_ reduction phase (Table 1).

CO selectivity (S_CO_) (Figure 6) is around 60 mol % for all the three composites, with an increase of about 20 mol % compared to the results reported in a previous work^[27]^ (Figure S8), for a commercial Cu‐based CO_2_ hydrogenation catalyst (CZA) physically mixed with the same mesostructured supports used in the present work. This difference can be primarily ascribed to a different activity of the two hydrogenation catalysts (CZA *vs*. CZZ); it can be indeed concluded that the CZZ catalyst, compared to commercial CZA, is more active towards the reverse water‐gas shift (RWGS) and/or methanol decomposition, according to the reactions:

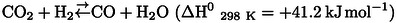




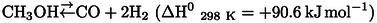




In this context, the selection of CZZ as the CO_2_ reduction phase for the nanocomposites was due to the fact that ZrO_2_ has a lower water affinity compared to Al_2_O_3_, thus leading to a higher water resistance of the Cu‐based reduction catalysts.[^19^, ^31^] The observed difference in terms of product selectivity between CZZ and commercial CZA could be thus ascribed to a different synthesis method, as well as to the presence of other phases (MgO,^[62]^ C)^[28]^ for the commercial catalyst.

Regarding DME selectivity (S_DME_) (Figure 6), it ranges from 3.4 mol % (CZZ@Al‐SBA‐15) to 11.3 mol % (CZZ@Al‐MCM‐41). Comparing these results with those shown by the same mesostructured aluminosilicates used in physical mixture with CZA,^[27]^ it can be noticed that, for the nanocomposites, DME selectivity shows an overall decrease, also following a different trend (Figure S8; Table S4). Particularly, in the present work S_DME_ follows the trend CZZ@Al‐MCM‐41>CZZ@Al‐SBA‐16>CZZ@Al‐SBA‐15, while in the mentioned reference^[27]^ the trend was Al‐SBA‐16+CZA>Al‐SBA‐15+CZA>Al‐MCM‐41+CZA. Provided that a direct comparison in terms of DME selectivity between the two series of catalysts is not straightforward, considering the different reaction conditions, CO_2_ hydrogenation species, and contact (nanocomposites *vs*. physical mixtures), the presence of two opposite trends still leaves space for some considerations.

The overall S_DME_ decrease for the composites can indeed presumably be attributed to the higher S_CO_ (both the RWGS and methanol decomposition negatively affect S_MeOH_ and, consequently S_DME_ production) but also to surface changes in the mesostructured acid supports due to the functionalization process with the hydrogenation phase. Particularly, it can be hypothesized that a fraction of the acid sites present on the surface of the supports, which are responsible for the dehydration of methanol to DME, is covered by the deposition of the CO_2_ reduction phase, thus being no longer able to adsorb and dehydrate methanol molecules.

Interestingly, CZZ@Al‐MCM‐41 shows the highest S_DME_ value among the nanocomposites, with a value (11.3 mol %) comparable to that observed for Al‐MCM‐41+CZA in^[27]^ (10 mol %), which, however, was the worst among the bare aluminosilicates. On the other hand, for the other two composites, a significant drop in terms of DME selectivity is observed, compared to.^[27]^ Particularly, CZZ@Al‐SBA‐15 shows a drop of 72 %, while a 79 % drop is found for CZZ@Al‐SBA‐16, compared to the corresponding supports mixed with CZA (Table S4). In the light of the above, the relatively high S_DME_ of CZZ@Al‐MCM‐41 can be attributed to its very high surface area that leads to a larger number of residual acid sites after the functionalization process. The significant difference with the other two support‐composite pairs can be ascribed to the difference in terms of surface area; particularly, CZZ@Al‐SBA‐16, despite being the most active dehydration catalyst, features the most drastic decrease, since it has the lowest surface area (Table 1).

As revealed by the microcalorimetric results, the deposition of the CO_2_ hydrogenation phase onto the surface of the supports leads to a decrement in the number of acid sites only in one case (Al‐SBA‐16 vs. CZZ@Al‐SBA‐16), while in the other two cases it gives rise to an increase in the total amount of acid sites (Figure 5, Table S3); this finding makes evident that a not simple correlation exists between the surface acidity and the selectivity to DME. However, it can be presumed that a number of Lewis acid sites from ZrO_2_ could be formed at the expense of the Brønsted ones as a consequence of the covering of the support surface during the functionalization process. As previously observed,^[28]^ indeed, Lewis acid sites show a very low activity towards methanol dehydration, due to their trend to deactivate due to water adsorption. On the other hand, the Brønsted sites of the aluminosilicates are much more active, being less sensitive to deactivation. Consequently, it can be assumed that the increase in the number of acid sites for two of the composites, observed with NH_3_‐microcalorimetry, does not correspond to an increase in terms of methanol dehydration activity due to the fact that the Brønsted sites of the support are partially covered with much less active Lewis sites. For this reason, NH_3_‐microcalorimetry does not provide information on the acid properties useful to explain the observed catalytic behavior. In order to perform a better correlation between the acidic properties and the catalytic behavior, a quantification of Brønsted and Lewis acid sites present on the nanocomposites would be useful. This characterization, previously performed using transmission FTIR spectroscopy with a probe molecule (*e. g*. pyridine) on the aluminosilicate supports,^[27]^ is unfortunately not applicable to the nanocomposite catalysts, due to their dark color, which does not allow to obtain a detectable signal.

Another comparison in terms of catalytic performance can be done by comparing the nanocomposite catalysts proposed in the present work with other nanocomposite catalysts synthesized in a previous work^[35]^ with the same self‐combustion approach on mesostructured γ‐Al_2_O_3_ supports (Figure S9). In that work, very low values of S_DME_ (<1 mol %) were observed due to the fact that, with respect to aluminosilicates, the mesostructured γ‐Al_2_O_3_ only featured the presence of Lewis acid sites, thus being less active towards methanol dehydration.[^27^, ^35^] Furthermore, the mesostructured γ‐Al_2_O_3_ supports used in that work have a lower surface area (~200 m^2^/g) compared to the aluminosilicates of the present work (437–1262 m^2^/g). In the light of the above, this comparison confirms that mesostructured aluminosilicates with Brønsted and Lewis acid sites and featuring a high surface area are much more appropriate acidic supports to host a CO_2_ reduction phase, with respect to mesostructured γ‐Al_2_O_3_.

As mentioned in the introduction, the number of works focusing on functionalized mesoporous/mesostructured systems for the synthesis of DME is very limited, since the vast majority of the papers focus on zeolites.[^19^, ^21^, ^63^] Furthermore, some of the reported functionalized mesostructured systems are used for the synthesis of DME from syngas (CO+H_2_),[^64^, ^65^, ^66^] rather than from CO_2_ and H_2_. Due to the significant thermodynamic differences between the two reactions,^[19]^ which allow the conversion of CO to reach much higher values compared with the conversion of CO_2_, a comparison between the results of the two reactions over similar catalysts is not appropriate. Table S5 reports the reaction conditions and catalytic performance of the catalysts reported in the present work with other composite systems, reported in the literature, consisting of a Cu‐based CO_2_ reduction phase supported on mesostructured/mesoporous oxides. Since the reaction conditions differ significantly, the results of the mentioned works are not completely comparable with the ones of this work, but they can be considered the most appropriate term of comparison with our results, due to the similar nature of the catalytic systems. Looking at the data reported in the literature (Table S5), it can be noticed that the catalytic performances of the nanocomposite catalysts presented in this work are comparable with those reported by the other authors, despite the comparison not being straightforward due to the different experimental conditions. Particularly, a significant effect of the GHSV on the values of CO_2_ conversion can be observed, as expected, where lower values of GHSV lead to higher values of X_CO2_. Indeed, the catalysts reported in^[70]^ show significantly higher values of X_CO2_ (22.0–24.5 mol %) compared to the present ones (3.4–5.6 mol %); this fact can be ascribed to the much lower GHSV used in^[70]^ (2000 cm^3^ g_cat_^−1^ h^−1^) compared to the one used in this work (12000 cm^3^ g_cat_^−1^ h^−1^). Besides the GHSV, a significant effect on X_CO2_ can be attributed to the fact that in^[70]^ a mesostructured γ‐Al_2_O_3_ support was used; in this context, γ‐Al_2_O_3_ could act not only as a dehydration catalyst but also as a promoter for the CO_2_ reduction catalyst. This assumption is supported by the fact that the nanocomposites reported by our research group in a previous work^[35]^ showed X_CO2_ values of 3.3–4.4 mol %, despite the GHSV value of 48000 cm^3^ g_cat_^−1^ h^−1^. The results of the present work and^[35]^ are also in line with those reported in,^[72]^ also in this case for γ‐Al_2_O_3_‐based catalysts; the X_CO2_ is slightly higher (3.9–7.4 mol %) than that reported in this work (3.4–5.6 mol %) and in^[35]^ (3.3–4.4 mol %) presumably due to the much lower GHSV used in^[72]^ (5000 cm^3^ g_cat_^−1^ h^−1^). On the other hand, DME selectivity of the catalysts reported in^[72]^ (1.2–7.3 mol %) is slightly lower than that of the present nanocomposites (3.4–11.3 mol %), probably due to the fact that aluminosilicates are more active towards methanol dehydration than γ‐Al_2_O_3_, as already mentioned. Finally, comparing the results of our aluminosilicate‐based composites with the catalysts reported in^[71]^ (based on SBA‐15) we can observe that our catalysts reach higher values of X_CO2_ (3.4–5.6 mol %) than those of^[71]^ (0.045–3 mol %). Again, this fact can be ascribed, at least in part, to the higher GHSV used in the reference work (30000 cm^3^ g_cat_^−1^ h^−1^). Besides the works mentioned in the table, it is worth noting that Atakan *et al*. also reported catalysts featuring a Cu‐based CO_2_ hydrogenation phase deposited on Zr‐SBA‐15;[^67^, ^68^, ^69^] however, their catalytic tests were performed in static conditions into a batch reaction cell, making a comparison not possible.

As a confirmation that an intimate contact between hydrogenation phases and acidic supports not necessarily promote the formation of DME, Bonura *et al*.^[31]^ observed very low performances for a bifunctional catalyst obtained from H‐ZSM‐5 and Cu/ZnO/ZrO_2_ with a coprecipitation method; the authors ascribed this behavior to a phenomenon of ion‐exchange between the acid sites of the zeolite and the metal cations of the CO_2_ hydrogenation phase, which caused the disappearance of the Brønsted sites of H‐ZSM‐5 with moderate strength. Due to this fact, the zeolite was left with strong sites only, unable to promote methanol dehydration.[^31^, ^32^] A detrimental effect of the intimate contact between the CO_2_ reduction catalyst and the acidic phase was also observed by other authors in the form of a performance drop over time for the syngas‐to‐DME process for H‐ZSM‐5 mixed with a Cu/ZnO/Al_2_O_3_‐based hydrogenation phase by kneading in a mortar followed by pelletization. This fact was ascribed to sintering phenomena of the CO_2_ hydrogenation phase particles, their oxidation and also to ion‐exchange of the hydrogenation phase with the zeolite, which led to a deactivation of the acid sites of the dehydration catalyst.^[33]^ A similar phenomenon, associated with a decrease in the amount of Brønsted acid sites, was also observed when the zeolite H‐ZSM‐5 was put into contact with the CO_2_ reduction catalyst through mechanical mixing (grinding) and liquid phase mixing (slurry), resulting in a drop in catalytic performance. On the other hand, this negative effect was not observed when the two catalysts were separately pelletized and subsequently mixed together.^[34]^ To summarize, in the present study, due to their significantly higher DME selectivity, all physical mixtures show, despite the lower X_CO2_ values, higher DME yields than their corresponding composites. These results, in accordance with what discussed above, could be explained assuming that the low activity of the CZZ catalyst allows the dehydration of methanol to prevail over its decomposition, allowing high selectivities towards DME.

## 3 Conclusions

In this work a study on nanocomposite bifunctional catalysts for the one‐step CO_2_‐to‐DME reaction, with different mesostructured supports, is presented. Particularly, three mesostructured aluminosilicates, namely Al‐MCM‐41, Al‐SBA‐15, and Al‐SBA‐16, were used as acidic supports to host a CuO/ZnO/ZrO_2_ (CZZ) CO_2_ reduction catalyst, with the aim of determining the influence of the textural properties of the supports on the growth and properties of the CO_2_ reduction phase. For this purpose, a synthesis route consisting in a self‐combustion reaction combined with an impregnation process was used, giving rise to a controlled growth of the reduction phase nanoparticles inside the mesoporous structure, as indicated by the lack of diffraction peaks in WA‐XRD patterns and by TEM and EDX measurements.

The catalytic performances of the nanocomposites for the CO_2_‐to‐DME reaction were determined and correlated with their structural and textural properties, as well as with those of their supports. The relatively high CO_2_ conversion of CZZ@Al‐SBA‐15 was attributed to its high pore volume and large pore size, which allow to obtain a high dispersion of the CO_2_ hydrogenation phase while avoiding the obstruction of part of the pores. Comparing the results with those of a previous work,^[27]^ in which the same mesostructured aluminosilicates were tested in physical mixture with a commercial CO_2_ reduction catalyst (CZA) for the same reaction, an influence of the reduction phase on product selectivity was observed. Particularly, the CZZ phase is responsible of an overall drop in terms of DME selectivity, associated with a rise in S_CO_. The S_DME_ decrease can be also associated with the functionalization with the CO_2_ hydrogenation phase, that causes a partial coverage of the acid sites of the support, deactivating them. In this context, an important effect associated with the surface area of the supports was observed. The CZZ@Al‐MCM‐41 nanocomposite, with the support featuring the highest surface area, does not show a decrease in S_DME_ compared to Al‐MCM‐41+CZA; on the other hand, the one with the support featuring the lowest surface area (CZZ@Al‐SBA‐16) shows the most significant drop (79 %). This finding can be ascribed to the higher fraction of Brønsted acid sites still uncovered and available for methanol dehydration in CZZ@Al‐MCM‐41, due to the high surface area of Al‐MCM‐41.

Starting from the data gathered in this work, future studies will focus on the optimization of the loading of CO_2_ reduction phase in nanocomposites; a lower loading should indeed lead to a higher amount of acid sites available for methanol dehydration and, thus, to a higher selectivity towards DME. As an alternative, pure siliceous mesostructures (MCM‐41, SBA‐15, SBA‐16) could be used as supports to disperse the reduction phase, obtaining supported CO_2_ hydrogenation catalysts; these catalysts would then be physically mixed with the mesostructured acidic systems. In this way, a high dispersion of the hydrogenation phase into a mesostructure would be obtained, while leaving all the acid sites of the acidic catalyst uncovered and available for methanol dehydration.

## 4 CRediT Authorship Contribution Statement

Fausto Secci: Conceptualization, Data curation, Formal analysis, Investigation, Methodology, Visualization, Writing – original draft. Valentina Mameli: Conceptualization, Data curation, Writing – review & editing. Marco Sanna Angotzi: Data curation, Investigation, Methodology. Luciano Atzori: Data curation, Investigation, Visualization, Writing – review & editing. Lorenza Piroddi: Formal analysis, Writing – review & editing. Nicola Pinna: Data curation, Writing – review & editing. Mauro Mureddu: Conceptualization, Methodology, Resources, Validation, Writing – review & editing. Carla Cannas: Conceptualization, Methodology, Visualization, Funding acquisition, Project administration, Supervision, Validation, Writing – review & editing.

## Conflict of Interests

The authors declare that they have no known competing financial interests or personal relationships that could have appeared to influence the work reported in this paper.

5

## Supporting information

As a service to our authors and readers, this journal provides supporting information supplied by the authors. Such materials are peer reviewed and may be re‐organized for online delivery, but are not copy‐edited or typeset. Technical support issues arising from supporting information (other than missing files) should be addressed to the authors.

Supporting Information

## Data Availability

The data that support the findings of this study are available from the corresponding author upon reasonable request.
