# Supplementary material for: Confined Growth by Self‐Combustion of a Cu‐Based Nanophase into Mesostructured Acid Supports for DME Production from CO2
Source: Chempluschem. 2025 Feb 4;90(5):e202400760. doi: 10.1002/cplu.202400760 (PMC12105452; doi:10.1002/cplu.202400760)
Supplement: Supplementary file 1 — Supporting Information [file CPLU-90-e202400760-s001.pdf]

ChemPlusChem

Supporting Information

Confined Growth by Self-Combustion of a Cu-Based Nanophase into Mesostructured Acid Supports for DME Production from CO_2

Fausto Secci, Valentina Mameli, Marco Sanna Angotzi, Luciano Atzori, Lorenza Piroddi, Nicola Pinna, Mauro Mureddu, and Carla Cannas*

Supporting information

Confined growth by self-combustion of a Cu-based nanophase into mesostructured acid supports for DME production from CO_2

Fausto Secci [a,b], Valentina Mameli [a,b], Marco Sanna Angotzi [a,b], Luciano Atzori [a,b], Lorenza Piroddi [c], Nicola Pinna [d], Mauro Mureddu [c], Carla Cannas [a,b]

[a] Department of Chemical and Geological Sciences, University of Cagliari, S.S. 554 bivio per Sestu, 09042 Monserrato, CA, Italy

[b] Consorzio Interuniversitario Nazionale per la Scienza e Tecnologia dei Materiali (INSTM), Via Giuseppe Giusti 9, 50121 Firenze, FI, Italy

[c] Sotacarbo S.p.A., Grande Miniera di Serbariu, 09013 Carbonia, SU, Italy

[d] Institut für Chemie and IRIS Adlershof, Humboldt-Universität zu Berlin, 12489 Berlin, Germany

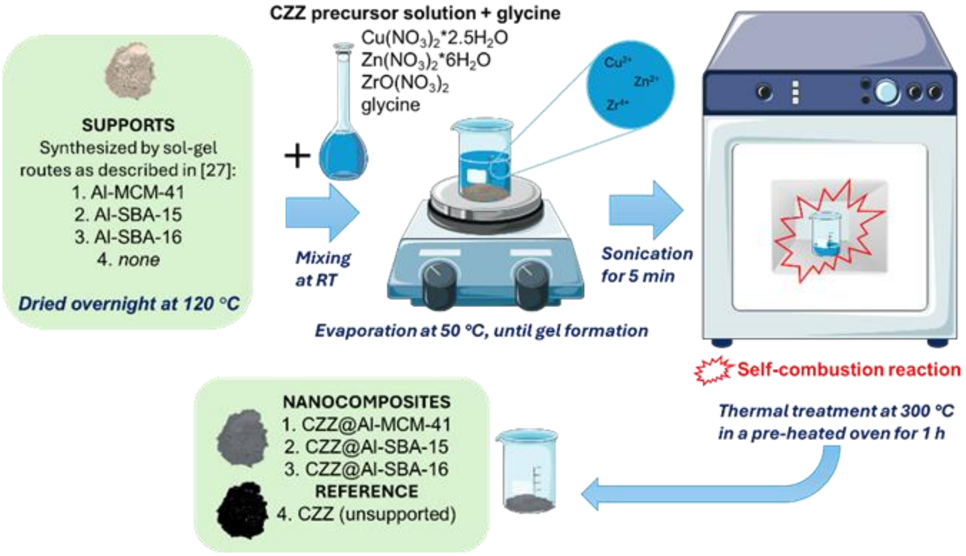

Figure S1 Sketch depicting the synthesis method based on sol-gel self-combustion process for the preparation of the CZZ-based nanocomposite catalysts by using mesostructured acid aluminosilicate catalysts as supports. For comparison, a reference sample (4) was also synthesized in the absence of the acid support through the same synthetic route.

Synthesis of CZZ hydrogenation catalyst (unsupported CZZ catalyst)

An unsupported $CuO/ZnO/ZrO_2$ (molar ratio 2/1/1.3) CO_2 hydrogenation catalyst, named CZZ, was synthetized by using the same self-combustion method based on a redox reaction between metal nitrates and glycine described in Paragraph 2.3 for the nanocomposite catalysts but without a support. Specifically, 50 mL of a nitrate-glycine solution prepared as described in paragraph 2.3 were heated into a beaker at 90 °C under stirring, in order to let water evaporate, until a blue viscous gel was obtained. The gel was then put into a pre-heated oven at 300 °C for 1 h, to ignite the self-combustion redox process. The beaker was eventually removed from the oven, observing the transformation of the blue gel into a black – dark brown powder.

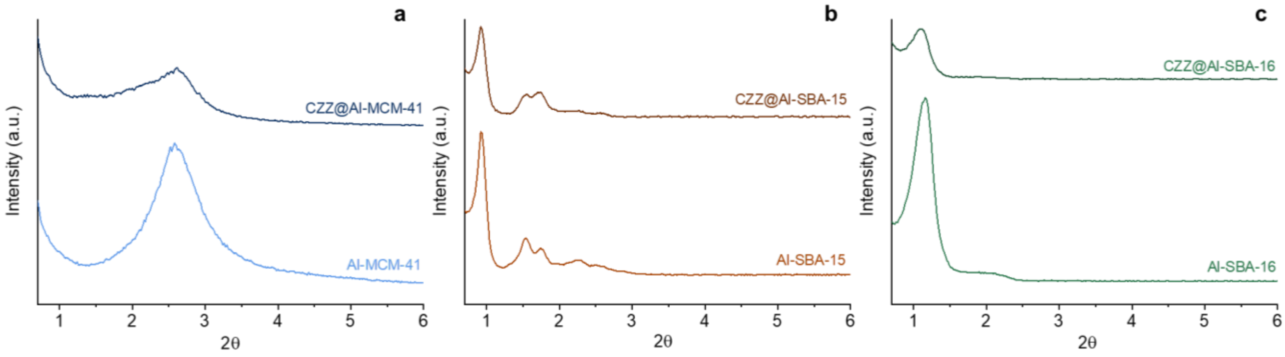

Figure S2 SA-XRD patterns of composites synthesized by self-combustion method on Al-MCM-41 (a), Al-SBA-15 (b), and Al-SBA-16 (c) compared with the corresponding mesostructured acidic supports. The patterns are reported with absolute intensity values.

Structural, textural, and morphological characterization of the unsupported CZZ catalyst

Figure 1b reports the wide-angle X-ray diffraction pattern of the CZZ hydrogenation catalyst obtained by a self-combustion procedure. The pattern clearly shows the presence of reflections attributable to different crystalline phases. The phase identification points out that

each atomic species of the CO_2 reduction catalyst (namely Cu, Zn and Zr) gave rise to the formation of phases with different crystal structure and different oxidation state. Particularly, Cu is present as tenorite (CuO, PDF card 00-045-0937), cuprite (Cu_2O, PDF card 00-005-0667) and, in low amount, as metallic copper (Cu, PDF card 00-004-0836); zinc is only present in its zincite form (ZnO, PDF Card 00-036-1451) and Zr is present as baddeleyite (monoclinic ZrO_2, PDF card 00-036-0420) and cubic ZrO_2 (PDF card 00-027-0997). A Rietveld refinement of the pattern (Figure S3) was performed in order to quantify the phases and the results of the analysis, reported in Table S1, are close to the theoretical 2/1/1.3 Cu/Zn/Zr molar ratio used for the synthesis. All phases but metallic copper formed as nanophases, in the 20-50 nm range of crystallite size, except for ZnO having average size of about 90 nm.

Table S1 Weight composition of the crystalline phases present in the unsupported CZZ CO_2 hydrogenation catalyst determined by Rietveld analysis.

**Phase**	**Cu**	**Cu_2O**	**CuO**	**ZnO**	**Monoclinic ZrO_2 (Baddeleyite)**	**Cubic ZrO_2**
**Amount (wt%)**	44.2			21.1	34.7	
	3.6(1)	21.6(6)	19.0(8)	21.1(7)	22.7(6)	12.0(3)
**Cell a (Å)**	3.620(1)	4.268(1)	4.676(4)	3.251(1)	5.151(2)	5.115(1)
**Cell b (Å)**	-	-	3.444(4)	-	5.205(2)	-
**Cell c (Å)**	-	-	5.123(7)	5.205(1)	5.317(1)	-
**Size (nm)**	>100	38(4)	19(1)	93(9)	41(2)	49(6)
**Microstrain**	0.0012(3)	0.0059(4)	0.0035(6)	0.0021(1)	0.0020(2)	0.0057(3)

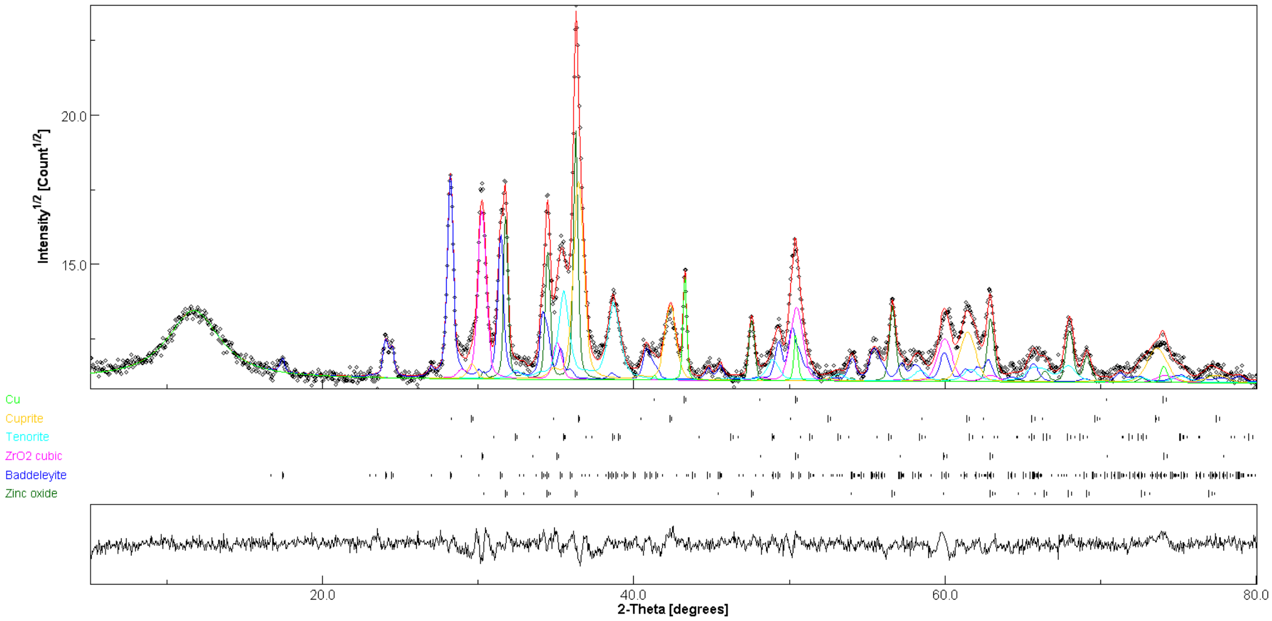

Figure S3 Rietveld analysis on the wide-angle XRD pattern of the unsupported CZZ CO_2 hydrogenation catalyst.

The unsupported CZZ catalyst present type II isotherms (Figure S4a), usually associated to non-porous or macroporous materials. This finding, associated with the very low values of surface area and pore volume (Table S2), indicates that the CZZ is presumably a non-porous material. This assumption is also confirmed by the very low values of pore volume reported in the BJH plot (Figure S4b); only a minor contribution can be observed below 4.5 nm, probably originated by a very low inter-particle porosity.

Table S2 BET surface area (SA) and pore volume (V_p) of the unsupported CZZ catalyst. Relative standard deviation: %RSD (SA) = 2.1 %; %RSD (Vp) = 1.1 %; %RSD (Dp) = 1.8 %.

Sample	SA (m^2/g)	V_p (cm^3/g)
**CZZ**	6.9	0.013

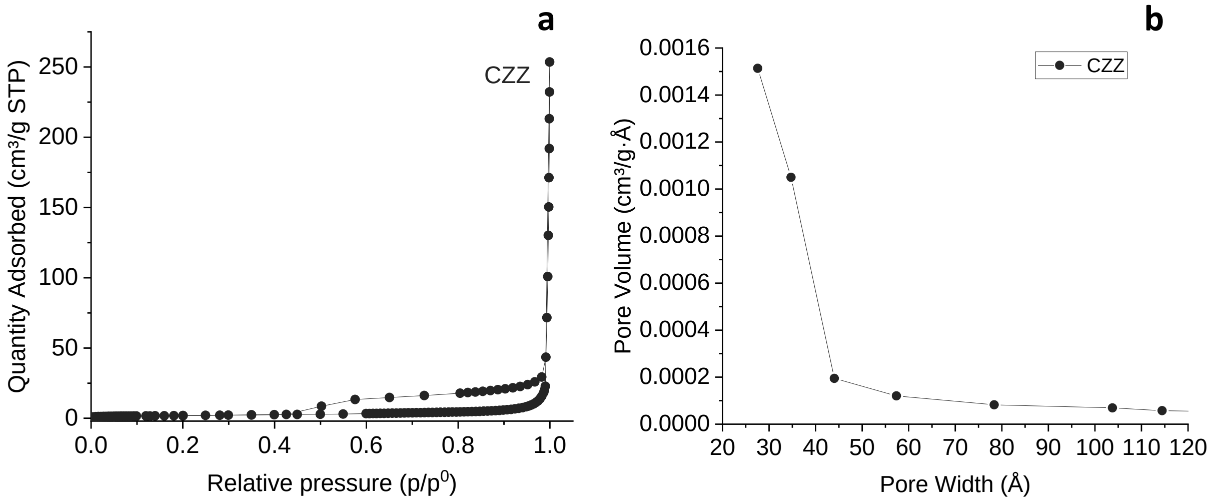

Figure S4 Nitrogen physisorption isotherms (a) and BJH pore size distribution (b) of the unsupported CZZ.

Figure S5 and Figure S6 depict the TEM, HR-TEM micrographs, and EDX chemical mapping images of unsupported CZZ CO_2 hydrogenation catalyst; some additional TEM micrographs are reported in Figure S7. As can be seen from TEM imaging, the unsupported CZZ catalyst consists of large particles of irregular shape and different appearance, presumably indicating significant differences in terms of composition. EDX chemical mapping also points out a clear separation of the different species associated to the CO_2 reduction phase (Cu, Zn, and Zr) indicating an inhomogeneous dispersion of different phases. HR-TEM analysis proves the phase separation of the oxide species present in the sample. Particularly, HR-TEM confirms the results obtained from EDX mapping; the crystal domains observed in HR-TEM micrographs, indeed, have been attributed determining their interplanar distance (d) and the determined phases correspond to the species observed from EDX mapping. From the correlation of HR-TEM with EDX analysis, it can be noticed how ZnO formed large particles with smooth edges; furthermore, these particles consist of either a few large or a single crystalline domain, in agreement with the Rietveld analysis which points out a mean crystallite size of 93(9) nm for ZnO. CuO, on the other hand, formed relatively small particles,

leading to the formation of large, poly-crystalline aggregates with irregular edges; these results agree with the mean crystallite size of 19(1) nm calculated by Rietveld analysis.

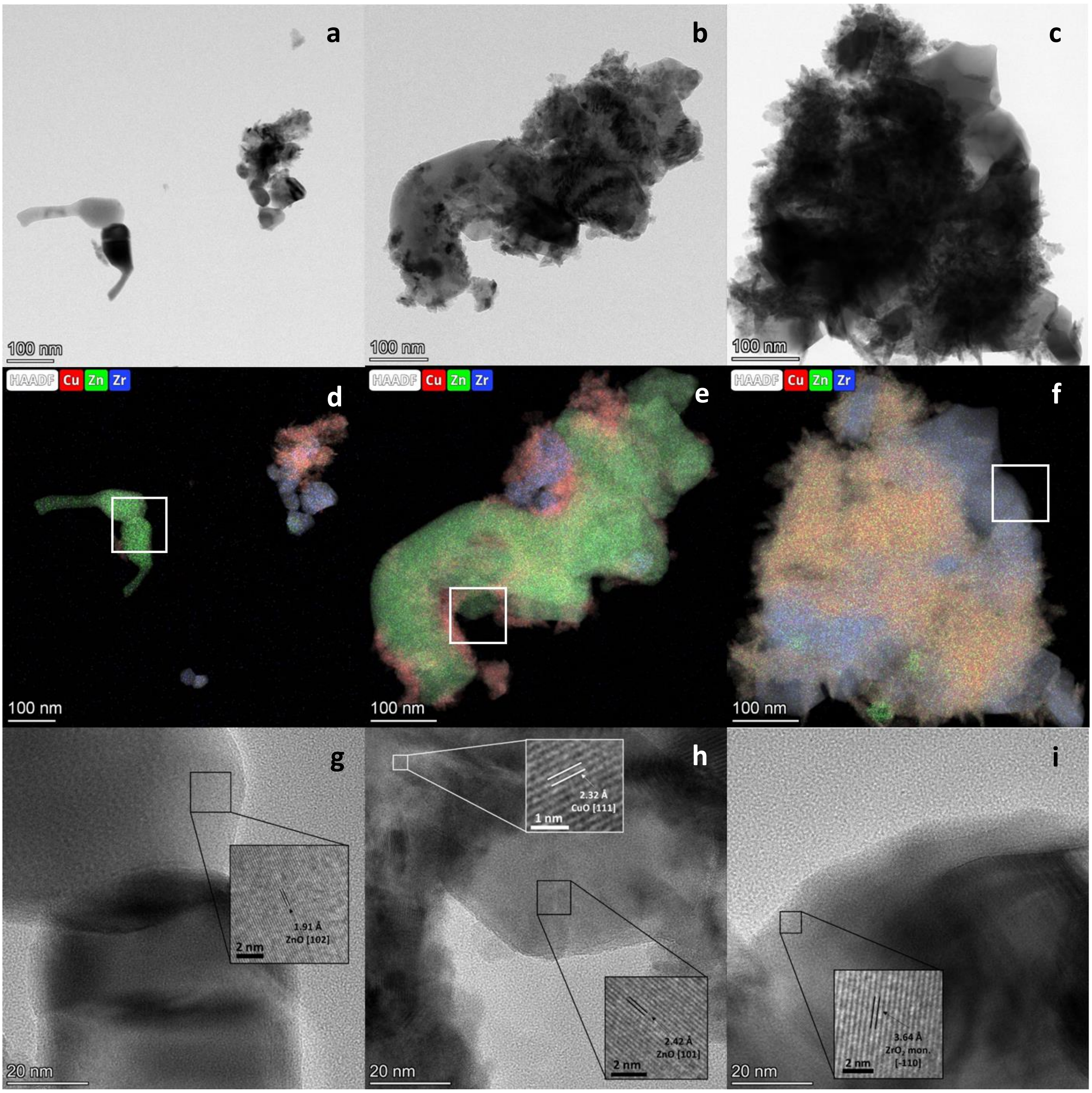

Figure S5 TEM micrographs (a-c), EDX chemical mapping (d-f), and HR-TEM micrographs (g-i) of the unsupported CZZ CO_2 hydrogenation catalyst.

The overall compositional inhomogeneity of the unsupported CZZ is also confirmed by line profile EDX (Figure S6). The elemental quantification performed with this technique clearly

shows different compositions over the investigated 2 μm linear site. Particularly, in this specific area, it can be noticed that Zn and Cu atoms are segregated in two different regions, and Zr is present in a very low amount.

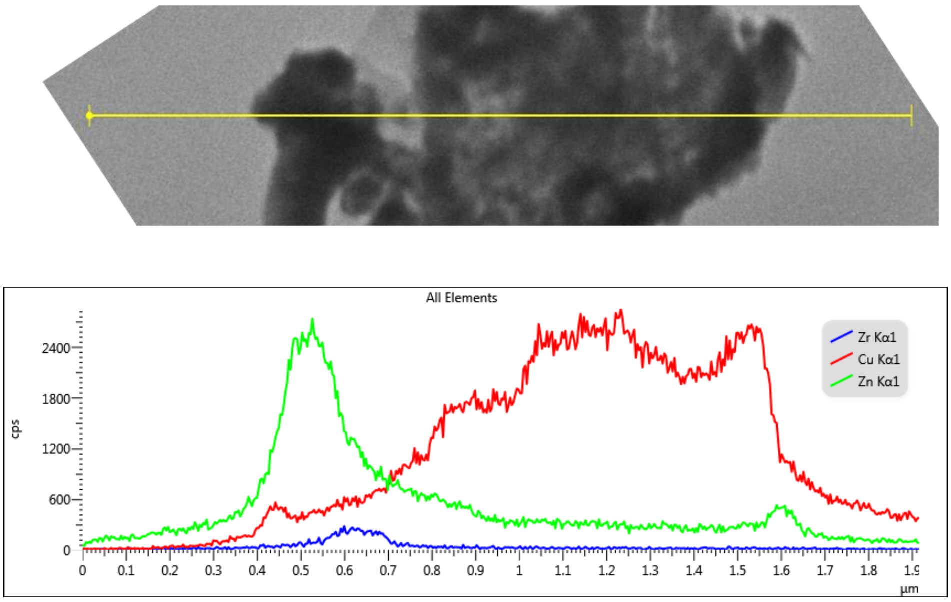

Figure S6 Line profile EDX analysis on CZZ.

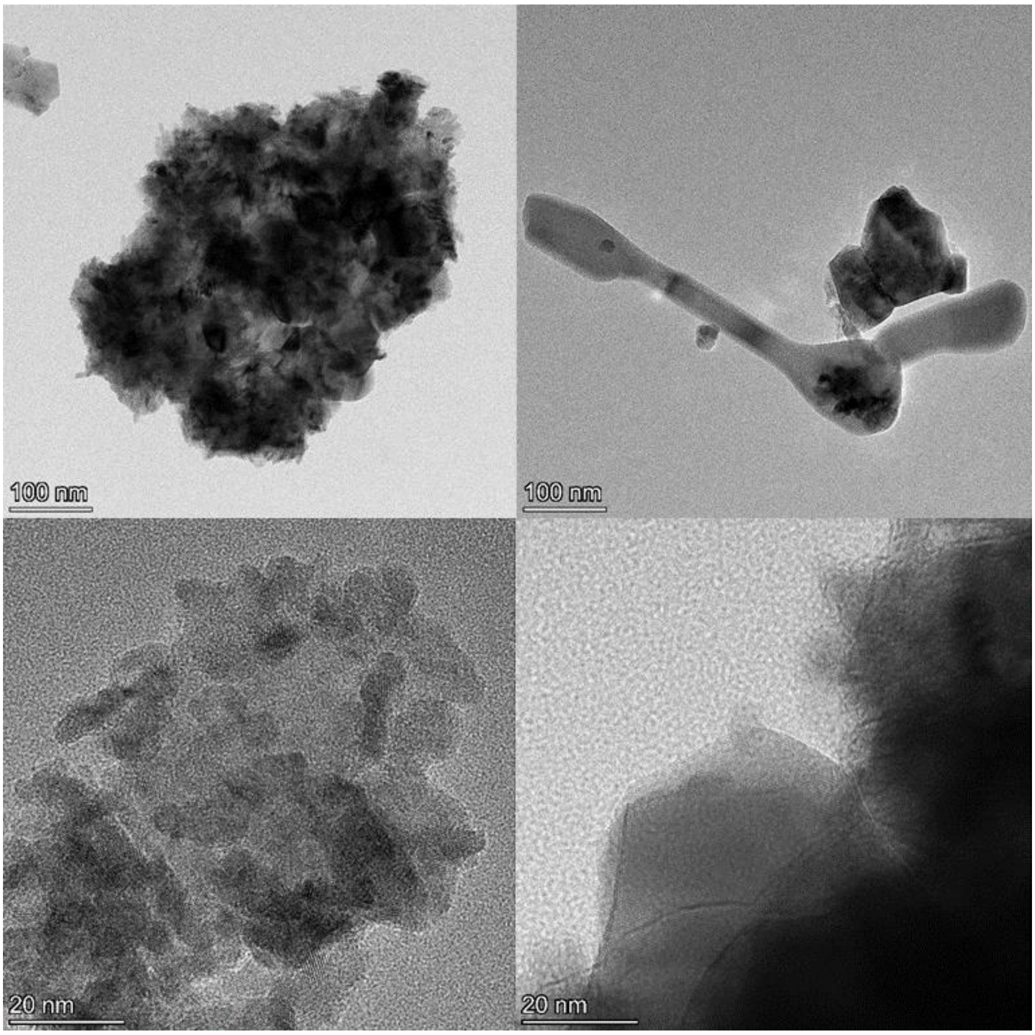

Figure S7 Additional TEM micrographs of the unsupported CZZ.

Table S3 Strength distribution of acid sites for CZZ, aluminosilicate supports, and composites.

**Sample**	**n_{NH_3} (µmol g^{-1})**			
	**$n_{NH_3,w}$[a]**	**$n_{NH_3,m}$[b]**	**$n_{NH_3,s}$[c]**	**$n_{NH_3,tot}$**
**CZZ**	6	-	-	6
**Al-MCM-41**	242	71	28	341
**CZZ@Al-MCM-41**	996	103	19	1118
**Al-SBA-15**	250	93	22	365
**CZZ@Al-SBA-15**	821	68	31	920
**Al-SBA-16**	218	87	35	340

**CZZ@Al-SBA-16**	200	61	25	286

Acid sites (n_{NH_3}): [a] 75 ≤ Q_{diff} < 110 kJ mol^{-1}; [b] 110 ≤ Q_{diff} < 150 kJ mol^{-1}; [c] Q_{diff} ≥ 150 kJ mol^{-1}; [d] Q_{diff} ≥ 75 kJ mol^{-1}.

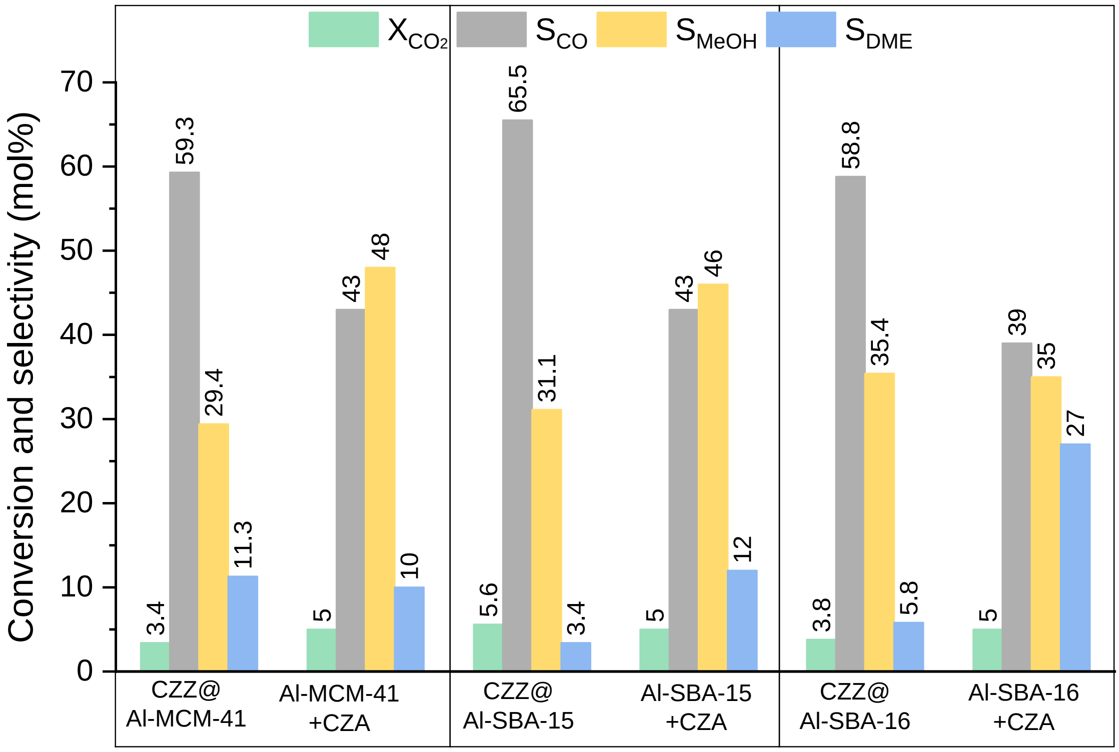

Figure S8 Mean values of CO_2 conversion and selectivity to CO, methanol and DME for the nanocomposites of the present work in comparison with those of the corresponding supports in physical mixture with a commercial CO_2 reduction catalyst (CZA) reported in [27].

Table S4 Mean values of CO_2 conversion and selectivity to CO, methanol and DME for the nanocomposites of the present work in comparison with those reported in [27].

**Sample**	**X_{CO2} (mol%)**	**Δ**	**S_{CO} (mol%)**	**Δ**	**S_{MeOH} (mol%)**	**Δ**	**S_{DME} (mol%)**	**Δ**	**Ref.**
**CZZ@Al-MCM-41**	3.4	-32%	59.3	+38%	29.4	-39%	11.3	+13%	This work
**Al-MCM-41 + CZA**	5		43		48		10		[27]
**CZZ@Al-SBA-15**	5.6	+12%	65.5	+52%	31.1	-32%	3.4	-72%	This work
**Al-SBA-15 + CZA**	5		43		46		12		[27]
**CZZ@Al-SBA-16**	3.8	-24%	58.8	+51%	35.4	+1%	5.8	-79%	This work

**Al-SBA-16 + CZA**	5		39		35		27		[27]

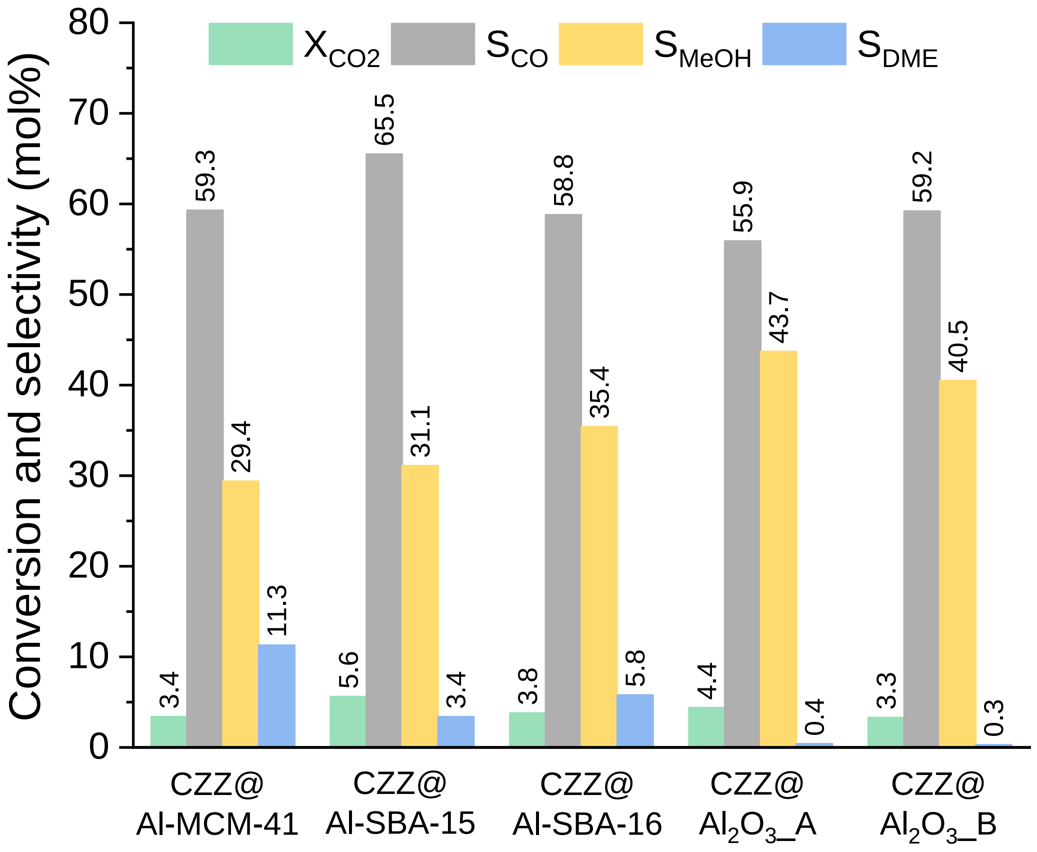

Figure S9 Mean values of CO_2 conversion and selectivity to CO, methanol and DME for the nanocomposites of the present work in comparison with those reported in [35].

Table S5 Catalytic results of the present work compared with other Cu-based mesostructured catalysts reported in the literature. The CO_2/H_2 molar ratio is 1/3 in all cases. The reduction phase weight % (Red wt%) is calculated considering the weight of the metal oxides.

Catalyst	T (°C)	P (MPa)	GHSV (cm^3 g_{cat}^{-1} h^{-1})	Red wt%	X_{CO2} (mol%)	S_{CO} (mol%)	S_{MeOH} (mol%)	S_{DME} (mol%)	Ref.
**CZZ@Al-MCM-41**	250	3	12000	33	3.4	59.3	29.4	11.3	This work
**CZZ@Al-SBA-15**	250	3	12000	33	5.6	65.5	31.1	3.4	
**CZZ@Al-SBA-16**	250	3	12000	33	3.8	58.8	35.4	5.8	
**CZZ@Al_2O_3_A**	250	3	48000	33	4.4	55.9	43.7	0.4	[35]
**CZZ@Al_2O_3_B**	250	3	48000	33	3.3	59.2	40.5	0.3	[35]
**Cu@Al_2O_3**	250	5	2000	12.5	22.0	48.0	38.9	12.6	[70]
**Cu@Ga-Al_2O_3**	250	5	2000	12.5	23.4	46.7	41.2	11.9	[70]

**Cu@Zn-Al_2O_3**	250	5	2000	12.5	24.5	40.8	45.2	13.2	[70]
**10Cu5Ga@SBA-15**	250	2.5	30000	15	3	<0.01	71	29	[71]
**4Cu2Ga@SBA-15**	250	2.5	30000	6	2	<0.01	73	27	[71]
**10Cu@SBA-15**	250	2.5	30000	10	0.045	<0.01	99.9	<0.01	[71]
**16Cu10Ga@SBA-15**	250	2.5	30000	26	1	<0.01	78	22	[71]
**17Cu13Zn@SBA-15**	250	2.5	30000	30	1	<0.01	99.9	<0.01	[71]
**Cu@Al_2O_3**	250	5	5000	12.5	6.4	79.9	18.8	1.2	[72]
**In(0.05)Cu@Al_2O_3**	250	5	5000	12.6	6.1	58.8	38.9	2.3	[72]
**In(0.1)Cu@Al_2O_3**	250	5	5000	12.6	7.4	51.4	44.6	4.0	[72]
**In(0.3)Cu@Al_2O_3**	250	5	5000	12.9	5.0	43.8	51.5	4.5	[72]
**In(0.5)Cu@Al_2O_3**	250	5	5000	13.1	3.9	29.7	62.5	7.3	[72]
